# Carvacrol—A Natural Phenolic Compound with Antimicrobial Properties

**DOI:** 10.3390/antibiotics12050824

**Published:** 2023-04-27

**Authors:** Wanda Mączka, Martyna Twardawska, Małgorzata Grabarczyk, Katarzyna Wińska

**Affiliations:** Department of Food Chemistry and Biocatalysis, Wrocław University of Environmental and Life Sciences, Norwida 25, 50-375 Wrocław, Poland; 121626@student.upwr.edu.pl

**Keywords:** carvacrol, antimicrobial, biotransformation

## Abstract

The main purpose of this article is to present the latest research related to selected biological properties of carvacrol, such as antimicrobial, anti-inflammatory, and antioxidant activity. As a monoterpenoid phenol, carvacrol is a component of many essential oils and is usually found in plants together with its isomer, thymol. Carvacrol, either alone or in combination with other compounds, has a strong antimicrobial effect on many different strains of bacteria and fungi that are dangerous to humans or can cause significant losses in the economy. Carvacrol also exerts strong anti-inflammatory properties by preventing the peroxidation of polyunsaturated fatty acids by inducing SOD, GPx, GR, and CAT, as well as reducing the level of pro-inflammatory cytokines in the body. It also affects the body’s immune response generated by LPS. Carvacrol is considered a safe compound despite the limited amount of data on its metabolism in humans. This review also discusses the biotransformations of carvacrol, because the knowledge of the possible degradation pathways of this compound may help to minimize the risk of environmental contamination with phenolic compounds.

## 1. Introduction

Carvacrol (5-isopropyl-2-methylphenol) is a monoterpenoid alcohol, a liquid with a boiling point of 236–237 °C and a density of 0.976 g/mL at 20 °C. It is insoluble in water but very soluble in ethanol, acetone, and diethyl ether [1]. It is a component of essential oils obtained from many plants, e.g., *Corido thymus*, *Lippia pepperwort* [2], black cumin (*Nigella sativa*), oregano (*Origanum compactum*), *O. dictamnus*, *O. microphyllum*, *O. onites*, *O. scabrum*, *O. vulgare*, pepperwort (*Lepidium flavum*), wild bergamot (*Citrus aurantium* var. bergamia Loisel), *Monarda didyma*, thyme (*Thymus glandulosus*), and savory (*Satureja hortensis*) [3]. It is worth noting that the content of carvacrol may vary depending on the tissue. In *Origanum vulgare* “Hot and Spicy”, carvacrol is the main component found in petals (94.40 ± 1.23%), tepals (96.92 ± 0.85%), bracts (96.07 ± 0.67%), and leaves (84.71 ± 1.59%), while *p*-cymene was dominant in the stems (65.44 ± 5.77%) and carvacrol was present in a minor amount (13.06 ± 6.74%) [4].

In plants, carvacrol is biosynthesized from γ-terpinene, which is formed by the mevalonate pathway [5] or by the plastid-based methylerythritol pathway (MEP) [6]. Recently, Krause et al. [7] proposed a carvacrol biosynthesis pathway in Lamiaceae plants in which γ-terpinene is oxidized by cytochrome P450 (CYP) monooxygenases of the CYP71D subfamily to produce an unstable cyclohexadienol intermediate. The resulting compound was then dehydrogenated by a short-chain dehydrogenase/reductase (SDR) to the corresponding ketone, which was converted to carvacrol. The combination of these enzymes produced carvacrol with γ-terpinene in both in vitro and in vivo tests in *Nicotiana benthamiana*. On the other hand, in the absence of SDR, no carvacrol formation was observed and only *p*-cymene was formed. Carvacrol’s biosynthetic pathway genes are highly co-expressed with transcription factor genes such as ZIP and basic helix–loop–helix (bHLH), indicating their involvement in the regulation of carvacrol biosynthesis [4]. CYP71D subfamily enzymes have also been detected in *Origanum vulgare* L. (CYP71D178, CYP71D180, and CYP71D181) [8]. In addition, Sun et al. [9] found genes in the *Thymus quinquecostatus* genome encoding terpene synthase (TPS), CYP, SDR, R2R3-MYB, and homeodomain–leucine zipper (HD-ZIP) IV. Researchers have shown that *Tq02G002290.1* (*TqTPS1*) encodes the terpene synthase responsible for the biosynthesis of γ-terpinene from geranyl diphosphate (GPP) [9]. TPS-encoding genes are found in *Origanum vulgare* (*OvTPS2*) [8], *Thymus vulgaris* (*TvTPS2*) [10], and *Thymus caespititius* (*TcTPS2*) [11]. The formation of γ-terpinene is also crucial in the synthesis of thymol—an isomer of carvacrol [9].

In turn, by organic synthesis, carvacrol is obtained as a result of Friedel–Crafts alkylation of *o*-cresol with propylene or isopropyl alcohol on solid acid catalysts (e.g., UDCaT-5, aluminum, or iron (III) chloride) [1,12]. Carvacrol can also be obtained by sulfonation of *p*-cymene followed by alkaline fusion [13], chlorination of α-pinene with *tert*-butyl hypochlorite [14], or as a result of the aromatization of carvone [12].

Carvacrol has been approved by the FDA for use in food and listed by the Council of Europe as a Category B chemical flavoring agent that may be added to foodstuffs at a level of 2 ppm in beverages, 5 ppm in flakes, and 25 ppm in candies [15]. Acute toxicity studies have been performed on a variety of animals, which is described in the next part of this publication.

Due to its flavoring (oregano-like smell and pizza-like flavor) [16] and antimicrobial properties, it is most often used in the food industry as a natural food preservative [17]. In dental practice, carvacrol has been used as a substitute for cretol and carbolic acid in the treatment of toothache, sensitive dentine, and alveolar abscess, and as an antiseptic in the pulp canals of the teeth [18]. It can also be used in the fight against mosquitoes, because it has much greater activity as a mosquito repellent than the commercial preparation, *N*,*N-*diethyl-*m*-methylbenzamide [19]. In addition, it reduces egg hatchability and induces infertility in mosquitoes.

**Figure 1:**
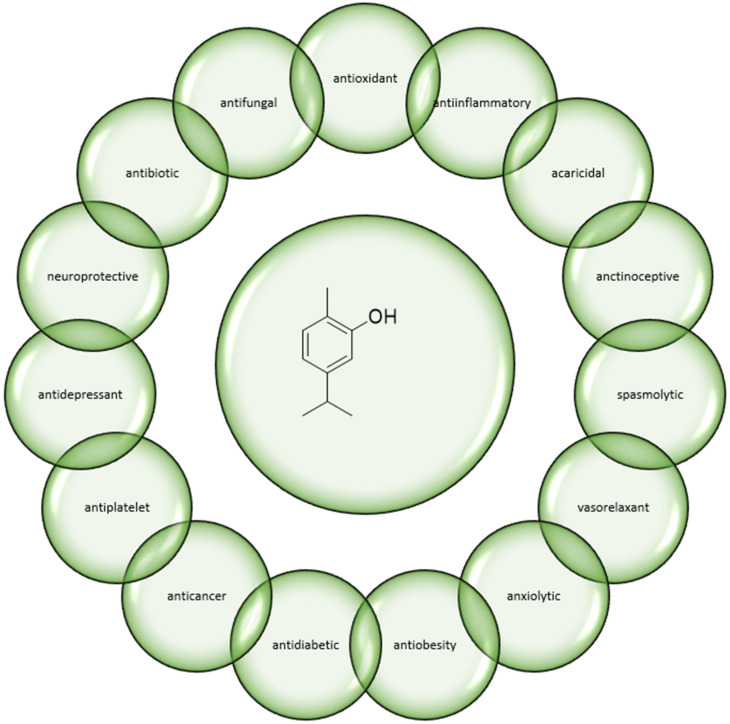
The broad spectrum of carvacrol activity.

In addition to antimicrobial properties, this compound also exhibits a wide range of other biological activities, including cardio-, reno-, and neuroprotective [20]; immune response-modulating [21]; antioxidant; anti-inflammatory [22]; anticancer [23,24,25]; analgesic [26]; anticonvulsant [27]; antidiabetic; hepatoprotective [28]; and anti-obesity properties [29,30] (Figure 1).

In the first part of our publication, the metabolism of carvacrol was discussed in detail, with particular emphasis on its effect on CYP. When discussing the antimicrobial properties, we paid attention to new forms of application of this compound. The challenges surrounding the wider use of carvacrol in food or feed are its unpleasant and pungent taste at higher doses; low bioavailability; high volatility; sensitivity to the surrounding environment, such as in processing conditions (e.g., heat or other ingredients); and the acidic environment in the digestive tract. The solution to the above problems seems to be the use of colloidal systems, including microencapsulation and nanotechnology, which were extensively discussed in the review by Wang and Wu [31]. Herein, we focused on the impact of these forms of application on the antimicrobial activity of carvacrol. Since infections are often accompanied by massive inflammation, an important advantage of an antimicrobial agent is its anti-inflammatory and antioxidant effects, which are discussed in the next part of our publication. We also called attention to the biotransformations that carvacrol can undergo in the environment, because the awareness of human impact on the environment is of great importance and it is essential not to contribute to the increase in pollution when introducing new products into the market.

## 2. Antimicrobial Activity of Carvacrol

Carvacrol has a strong antimicrobial effect against many strains of bacteria and fungi that are dangerous to humans or cause significant losses in the economy (Table 1). A strain of *Candida albicans*, next to *C. glabrata*, *C. krusei*, *C. tropicalis*, and *C. parapsilosis*, is one of the few species of fungi that causes diseases in humans [32]. In people with a healthy immune system, *C. albicans* is often harmless, remaining in balance with other members of the local microflora and asymptomatically colonizing the digestive tract, reproductive system, mouth, and skin. However, changes in the host microbiota (e.g., due to antibiotic intake), changes in the host’s immune response (e.g., during stress, infection with another microorganism, or immunosuppressive therapy), or changes in the local environment (e.g., changes in pH or nutrients) may allow for excessive growth of *C. albicans* and cause infection. These infections range from superficial mucosal and skin infections, such as thrush, vaginal yeast infections, and diaper rashes, to blood-borne disseminated infections, which have a high mortality rate. Infections with this pathogen account for 15% of all cases of hospital-acquired sepsis and are particularly serious in immunocompromised individuals (e.g., those with AIDS or those undergoing cancer or immunosuppressive therapies) and those with implanted medical devices [33]. This strain of *C. albicans* produces highly structured biofilms composed of multiple cell types encased in an extracellular matrix. It is worth noting that fungal biofilms are largely resistant to current antifungal drugs, so high doses are generally required to treat infections [34]. In the research by Jafri et al. [35] and Niu et al. [36], *C. albicans* cells treated with carvacrol showed irregular surfaces, multiple hyphal lesions, reductions in cell number, inhibition of biofilm formation by up to 80%, and inhibition of ergosterol synthesis. In turn, Miranda et al. [37] tested the activity of carvacrol against *C. albicans*, *C. glabrata*, *C. krusei*, and *C. dubliniensis*, confirming the antifungal activity of this compound from MIC 161.3 mg/L.

Ismail et al. [38] undertook research on the activity of carvacrol against the pathogenic *Candida auris* strain. They showed that the growth of all *C. auris* isolates was inhibited by carvacrol in the MIC range of 125–500 μg/mL, and the MFC values for the same isolates were in the range of 250–1000 μg/mL. *C. auris* antioxidant enzymes such as catalase (CAT), superoxide dismutase (SOD), glutathione peroxidase (GPx), glutathione reductase (GR), and glutathione transferase (GST) were analyzed and evaluated after exposure to carvacrol. It was observed that exposing the cell to increasing concentrations of carvacrol resulted in an increase in the activity of CAT, SOD, and GPx. In contrast to GR and GST, there was an obvious decrease in activity after exposure to carvacrol. The authors of the study proved that carvacrol is able to reduce the expression of genes encoding antioxidant enzymes. They also confirmed the effect of carvacrol on hemolysis in the range of 0.9–26.8%.

The antifungal activity of carvacrol in combination with antibiotics such as fluconazole, ketoconazole, or amphotericin B was tested against eight strains of *Candida*, including *C. tropicalis*, *C. parapsilosis*, *C. guilliermondii*, and *C. krusei*. The MIC for carvacrol alone ranged from 128 to 512 μg/mL. Different forms of interactions between carvacrol and antifungal agents have been observed: synergism in 25% of the combinations, additivity in 25%, and, finally, inertness in 50% of the combinations. No antagonist effect was observed. A synergistic effect was observed with the combination of carvacrol/ketoconazole and Amphotericin B against *C. guilliermondii* LM-103 and carvacrol/fluconazole against *C. parapsilosis* ATCC 22019 [39].

Regarding the wider use of carvacrol as an antimicrobial agent, its bioavailability is a major limitation. To this end, Mauriello et al. [40] encapsulated carvacrol using various stabilizers, such as whey protein isolate and surfactant Tween 80, and measured its antimicrobial activity against *Saccharomyces cerevisiae*. The obtained results indicated antifungal activity at a carvacrol concentration of <500 mg/L. Pure carvacrol had a stronger fungicidal effect than the Tween 80 emulsion containing 25% and 50% carvacrol. When whey protein isolate was used, the antimicrobial activity was proportional to the dissolution of carvacrol, suggesting that the emulsion droplets act as micrometric reservoirs for the compound, which is gradually released in the aqueous phase. Researchers postulate that the use of encapsulation may also contribute to extending its durability.

A certain strain of *Aspergillus flavus* is a common contaminant in grain, oil, and their products. Its metabolite, aflatoxin B1 (AFB1), has been proven to be highly carcinogenic. Therefore, it is very important to find antifungal substances that could inhibit the growth and production of toxins by *A. flavus* [41]. The optimal pH for the growth of the eight toxigenic Aspergillus strains was reported to be pH 6 in unmodified potato dextrose broth. Carvacrol, at 1.0 mM, completely inhibited fungal growth, but only at pH 4 and 8. At lower concentrations (0.1 and 0.5 mM), partial inhibition of mycelial growth was observed at all pH levels, with significantly less mycelial growth at pH 4 and 8 [42]. Qu et al. [43] found that carvacrol was effective in inhibiting *A. flavus* growth and AFB1 production. Carvacrol, when used at concentrations of 0, 50, 100, and 200 μg/mL, inhibited spore germination, mycelial growth, and AFB1 production in proportion to the concentration. In studies on the mechanism of action of carvacrol against *A. flavus*, it was found that the production of ergosterol (5,7-diene oxysterol) in the mycelium decreased with the increasing concentration of carvacrol [43]. Ergosterol is synthesized in the endoplasmic reticulum through the sequential activity of 25 different enzymes [44]. It is important for the structure of the cell membrane because its absence causes defects in the integrity of the cell membrane of the microorganism and an imbalance in cell permeability [45,46], which, in turn, can lead to eventual cell death. Thus, carvacrol was able to damage the cell membrane. The effect of carvacrol on *A. flavus* lipids was also investigated using a lipidomic metabolism analysis. When carvacrol was used at 200 µg/mL, a decrease in the level of most glycerolipids and phospholipids in the mycelium of *A. flavus* was observed. Neutral lipids decreased, as did phosphatidylcholines, phosphatidylethanolamines, and phosphatidylserines, while phosphatidylinositol and phosphatidylglycerols increased [43]. Glycerophospholipids are key components of the cellular lipid bilayer, and the composition of fatty acids has a significant impact on properties of the cell membrane such as its fluidity or its transport of triacylglycerols and cholesterol. Proper glycerophospholipid metabolism is essential for optimal cell membrane dynamics [47]. Therefore, external factors that induce changes in the amount of certain types of glycerophospholipids can affect the overall cellular metabolism [43].

Carvacrol placed on nanofibers is used to produce packaging. Fonseca et al. [48] conducted an experiment using nanofibers containing starch and carvacrol. Carvacrol alone contributed to a greater inhibition of growth on day 7 by 13% of *Penicillium* sp. and by 30% of *A. flavus* ATCC 204304. The MIC values for both carvacrol and nanofibers depended on the concentration. It is worth emphasizing that both Penicillium and Aspergillus fungi are responsible for food spoilage, and, thus, an active antifungal agent is being sought for use in packaging that will effectively limit the growth of these microorganisms and, at the same time, will not pass into food. The use of nanofibers with the addition of carvacrol seems to be a promising alternative to the currently used solutions.

Carvacrol was also active against plant soilborne pathogens (*Alternaria tomatophila*, *Podosphaera xanthii*, and *Xanthomonas perforans*) which are common in South Florida of the United States [49].

The antifungal activity of carvacrol was also tested on strains of Hyphomycetes (*Alternaria solani*, *Botrytis cinerea*, *Fusarium oxysporum*, *Pyricularia grisea*, and *Rhizoctonia solani*) and Oomycetes (*Phytophthora capsici* and *Phytophthora nicotianae*). Carvacrol, at a dose of 50 µg/mL, was active against the above strains, with a degree of inhibition greater than 90% against *B. cinerea*, which was equal to the commercial fungicide chlorothalonil [50]. The search for an effective antimicrobial agent is particularly important for *B. cinerea*, as it affects more than 1400 plant species, including many economically important ornamentals, fruits, and vegetables. It causes stem and fruit rot both before and after harvest. The economic losses caused by this phytopathogen are estimated to be between USD 10 and 100 billion worldwide [51]. The MIC for carvacrol was 120 mg/L, and the MBC was 140 mg/L. It changed the morphology of *B. cinerea* hyphae by disrupting and distorting the mycelium. It also negatively affected cell membrane permeability and caused a marked decrease in the total lipid content in *B. cinerea* cells, suggesting that cell membrane structures were destroyed [52].

On the other hand, harvesting fresh citrus fruits in warm and humid regions such as Florida destroys *Lasiodiplodia theobromae*, which causes Diplodia stem-end rot (SER). The EC_50_ value was 0.045 mg/mL. Carvacrol and thymol were then incorporated into a commercial shellac coating and applied to “Ruby Red” grapefruit inoculated with *L. theobromae* to determine their activity against Diplodia SER in vivo. Fruits were artificially inoculated with *L. theobromae* 12 h before or immediately after coating, then incubated at 29 °C and 90% relative humidity for 48 h. Lesion development was inhibited more effectively when a coating containing 10 mg/mL carvacrol was applied prior to pathogen inoculation. In addition, the inclusion of carvacrol in the shell did not adversely affect fruit weight loss, skin color, or titrated acidity [53].

Bacteria such as *Enterobacter cloacae*, *Escherichia coli*, *Listeria monocytogenes*, *Pseudomonas fluorescens*, *Pseudomonas putida*, and *Staphylococcus aureus* often develop during meat storage. Eating meat contaminated with bacteria can be dangerous to human health. Therefore, work is underway to develop packaging that would effectively inhibit the growth of microorganisms. Xiao et al. [54] obtained a film containing chitosan, pullulan, and carvacrol, which had acceptable technical parameters such as water vapor permeability, tensile strength, and percentage elongation at break. At the same time, its use allowed the shelf life of goat meat to be extended to more than 15 days. Another solution was to incorporate carvacrol and carboxybetaine into the polyurethane, slowing down the release of carvacrol from the material. A synergistic antibacterial effect of bio-derived “kill and gun” polyurethane against *E. coli* and *S. aureus* was observed [55].

On the surface of materials commonly used in medicine and food production, a microbial biofilm often develops which is difficult to remove and promotes the development of pathogenic bacteria. Carvacrol inhibited biofilm formation by up to 74–88% for *Pseudomonas aeruginosa* ATCC15442, and up to 86–100% for *S. aureus* ATCC6538 [56].

An interesting solution seems to be the combination of carvacrol with proteolytic enzymes. Mechmechani et al. [57] found a synergistic effect of a combination of carvacrol with pepsin and trypsin on the degradation of *P. aeruginosa* and *E. faecalis* biofilms growing on polystyrene or stainless steel surfaces. The effect was best after sequential treatment with two enzymes prior to administration of carvacrol. The enzymes were effective in destroying biofilms without causing cell death. By interacting with proteins, they caused structural defects in biofilms, thus worsening the barrier properties.

A strain of *Streptococcus pyogenes* (group A streptococcus (GAS)) is a Gram-positive bacterium commonly associated with pharyngitis. However, it can also cause scarlet fever, impetigo, cellulitis, type II necrotizing fasciitis, streptococcal toxic shock syndrome, acute rheumatic fever, and post-streptococcal glomerulonephritis. Approximately 18.1 million people currently suffer from the serious disease GAS, with 1.78 million new cases and 500,000 deaths each year [58]. A strain of *S. pyogenes* has the ability to form a biofilm to evade host defense systems. The biofilm is formed by microbial cells that are irreversibly bound to a substrate or interface, or to each other. They are encapsulated in the EPS matrix which they produce, and exhibit an altered phenotype in terms of growth rate and gene transcription. In the studies of Wijesundar et al. [59], it was found that carvacrol induced deformations and damage to the bacterial cells, as well as deactivation of extracellular polymers (EPS), which, in the bacterial cell, are responsible for protecting the cell against toxic substances and ensuring its strength. In addition, carvacrol reduced the hydrophobic properties of multicellular bacterial structures by up to 84.2%, and was able to reduce the expression of the *LuxS* gene associated with the formation of biofilms by *S. pyogenes* [59]. Carvacrol also resulted to be a promising antifungal agent against *S. aureus* and *S. epidermidis*, which mainly cause diseases of the skin and the respiratory, urinary, and digestive systems, as well as osteomyelitis of the bone and meningitis. In a study by Mauriello et al. [40], the authors attempted to correlate carvacrol with whey emulsion and compared the effect of pure carvacrol with that of an emulsion. The obtained results indicated a strong bactericidal effect against *S. epidermidis* ATCC 12228 and *P. fluorescens* ATCC 13525 at a carvacrol dose of <500 mg/L. In turn, Luna et al. [60] used nanotechnology, which increased the chemical and physical stability of carvacrol, extended its duration, and increased its effectiveness. For the synthesis of carvacrol-delivering nanoparticles by ionotropic gelation, they used chitosan due to its biocompatibility, biodegradability, intrinsic antibacterial activity, and hemostatic and mucoadhesive properties. Its activity against *E. coli* ATCC 25922 and *S. aureus* ATCC 25923 was tested, and it showed higher antibacterial activity compared to nanoparticles. However, it is worth noting that the total amount of carvacrol released from the nanoparticles after 24 h was significantly lower than that of the free chemical. In turn, Cui et al. [61] obtained a nanomaterial based on cationic starch nanofibers containing carvacrol and casein nanoparticles, and studied the effect of this material on the presence of *Bacillus cereus* in soy products. It was found that the obtained nanofibers were able to effectively inhibit the growth of *B. cereus* during cold storage and to maintain the sensory qualities of soybean products.

In turn, Mechmechani et al. [62] investigated the effect of microcapsules of carvacrol on the biofilm-forming capacity of *P. aeruginosa* and *Enterococcus faecalis*. Carvacrol was able to reduce the *P. aeruginosa* biofilm to below the limit of detection after only 15 min. A 4-fold lower MIC (1.25 mg/mL) than free carvacrol was observed for *P. aeruginosa* (MIC = 5 mg/mL) when microcapsules were used. However, in relation to *E. faecalis*, comparable results were obtained for both free carvacrol and microcapsules (MIC = 0.625 mg/mL). Carvacrol destabilized the bacterial cell membrane, leading to cell death.

The effect of carvacrol in the form of nanocapsules on the formation of a biofilm by *Salmonella* Enteritidis adhering to stainless steel was investigated by Yammine et al. [63]. Spherical nanocapsules with sizes of 159.25–234.76 nm were obtained using spray drying. Although, in the case of free carvacrol, the MIC was present at the level of 1.25 mg/L, after the application of this compound in the form of nanocapsules, the MIC decreased to 0.31 mg/L. It is worth noting that the MIC of thymol in the form of nanocapsules was 0.62 mg/L. The anti-biofilm activity of free and nanoencapsulated carvacrol was dose-dependent. Elimination of *S.* Enteritidis biofilms developed on stainless steel was achieved after 15 min of treatment with carvacrol nanocapsules at twice the MIC dose. Carvacrol, in the form of nanocapsules, showed no toxicity to *Daphnia magna* crustaceans after 48 h of exposure.

In addition, Fang et al. [64] determined that the MIC values for carvacrol against *Vibrio parahemolyticus* ATCC 17802, *Shewanella putrefaciens* ATCC 49138, *S. aureus* ATCC 6538, and *P. fluorescens* ATCC 13525 were 0.5, 0.5, 0.125, and 0.5 mg/mL, respectively. Carvacrol was found to alter cell membrane permeability, causing nucleic acids and proteins to leak and resulting in cell death. This mechanism was confirmed by the observation of alkaline phosphatase (AKP) leakage. This is an intracellular enzyme located between the cell wall and the cell membrane, and in the event of damage to the cell wall, there is a strong leakage of AKP outside the bacteria into the extracellular environment. The same researchers also obtained a carvacrol/β-cyclodextrin emulsion, which they applied to edible films of flaxseed gum (FSG)-sodium alginate (SA) to test the application of such a system for storing Chinese sea bass (*Lateolabrax maculatus*) fillets. The addition of carvacrol at a concentration of 1.0 mg/L ensured excellent organoleptic properties during cold storage of the meat. However, carvacrol at 2.0 mg/L imparted a strong characteristic taste, which is not desirable [64].

Carvacrol was also effective against multidrug-resistant *Klebsiella pneumoniae*, which is responsible for the development of pneumonia, as it eliminated all bacterial cells within 4 h. It showed low MIC and MBC values (ranging from 130 to 260 mg/L) for *K. pneumoniae* strains resistant to carbapenems and polymyxins. The obtained results encouraged the researchers to test its effectiveness in a mouse model of pneumonia. Administration of carvacrol to mice (10, 25, 50 mg/kg) was associated with increased survival and significantly reduced bacterial load in peritoneal washings. In addition, the carvacrol-treated groups had a significant reduction in the total white blood cell count and a significantly increased platelet count compared to the group of mice not given carvacrol [65].

In addition, Khan et al. [66] studied the activity of carvacrol against *E. coli* producing extended-spectrum β-lactamases (ESBLs) that were isolated from the blood of patients with urinary tract infections. Carvacrol, which has a minimum inhibitory concentration of 150 μg/mL and a minimum bactericidal concentration of 300 μg/mL, reduced the number of *E. coli* cells in a time-dependent manner. In addition, carvacrol was found to cause greater membrane depolarization and increased oxidative stress in *E. coli* cells; it also induced the release of cellular DNA, proteins, and potassium ions from bacterial cells and reduced both the quantity of bacteria in macrophage invasion assays and the levels of the inflammatory proteins TNF-α and COX-2. It has also been verified that carvacrol inhibits the activity of β-lactamase.

Carvacrol also inhibited the growth of *Dickeya zeae* (formerly *Erwinia chrysanthemi* pv. *zeae*), which causes huge economic losses because it attacks banana, rice, maize, and potato crops. Its presence has been noted in the USA, Asia, Australia, Africa, and Europe. The MIC (0.1 mg/mL) and MBC (0.2 mg/mL) values were determined against the *D. zeae* strain MS1, which was isolated from banana stems with soft rot symptoms. In addition, carvacrol damaged the cell membrane, as a reduction in membrane potential, a decrease in ATP concentration, and nucleic acid leakage were found. In addition, at sub-inhibitory concentrations, a significant inhibition of the swimming motility and biofilm formation of *D. zeae* MS1 was observed. A tissue infection test was also performed, showing that carvacrol significantly reduced the pathogenicity of *D. zeae* MS1. Inoculated banana seedlings showed significantly fewer disease symptoms after treatment with carvacrol, and the effectiveness against banana soft rot was 32.0% 14 days after inoculation [67].

Kasthuri et al. [68] tested the activity of carvacrol against *S. aureus* ATCC 6538, *P. aeruginosa* ATCC 15442, *E. coli* ATCC 10536, and *Enterococcus hirae* ATCC 10541 according to the protocol of the British Standard European Norm 1276: phase2/step1 (EN1276). In addition, a synergistic effect of the combination of carvacrol and nerol in the proportions of 0.625% carvacrol + 1.25% nerol and 0.781% carvacrol + 1.56% nerol was found. In addition, this combination destroyed the pre-formed biofilm. The effect of these terpenes on benign keratinocyte (HaCaT) cells was also tested, and no toxic effects were found. On this basis, the researchers postulate that the above combinations of carvacrol and nerol may be active ingredients in preparations for washing and disinfecting the hands of people in both hospital and home environments.

The synergistic effect of carvacrol with other terpenoids was also found by Sousa et al. [69] against *Gardnerella* sp. In bacterial vaginosis (BV), an increase in anaerobic bacteria is observed, leading to the formation of a multimicrobial biofilm consisting mainly of *Gardnerella* sp. BV is usually treated with broad-spectrum antibiotics such as metronidazole and clindamycin, resulting in a high number of relapses due to the simultaneous eradication of the beneficial *Lactobacillus* strains as well. For this reason, new alternative treatments are sought. Carvacrol in combination with *p*-cymene showed strong synergistic antimicrobial activity against *Gardnerella* sp. planktonic cultures. For biofilm elimination, the combination of carvacrol and linalool at concentrations below the MIC was found to be effective without exhibiting the cytotoxicity observed in reconstituted human vaginal epithelium [69].

A synergistic effect of carvacrol and nisin was found against *Listeria monocytogenes* and *S. aureus* BNCC 186335 strains. For *S. aureus* BNCC 186335, the combination of carvacrol and nisin resulted in a FICI of 0.281 and an FBCI of 0.09375. In addition, a decrease in biofilm formation was observed. Nisin facilitates the entry of carvacrol into the cell. The combination of carvacrol and nisin reduced the number of microorganisms growing in pasteurized milk and maintained the milk quality at 25 °C and 4 °C [70]. Carvacrol alone gave a MIC of 250 μg/mL and an MBC of 250 to 500 μg/mL when tested on 9 strains of *L. monocytogenes*. Bacterial cells exposed to carvacrol showed depolarization of the cell membrane and its increased permeability and changes in respiratory activity. The effectiveness of the combination of carvacrol and nisin was tested by storing sliced Bolognese sausages at 4 °C. A significant reduction in the growth rate of *L. monocytogenes* was then observed compared to the controls [71]. This bacterium is able to survive and multiply at refrigerator temperatures and is ubiquitous in the environment, easily contaminating vegetables, fruits, dairy products, meat, seafood, and ready-to-eat foods. It causes listeriosis in susceptible individuals, including the elderly, the immunocompromised, and pregnant women, with an overall mortality rate of 20–30%, depending on the country [72].

A synergistic effect against *E. coli* CICC 10664 and *S. aureus* CICC 21600 was also found for the combination of ε-poly-L-lysine (ɛ-PL) with carvacrol [73]. ε-PL is a polymer consisting of a series of 25–35 L-lysine monomers that is derived from metabolites produced by fermentation by *Streptomyces albus* 346 [74]. ε-PL has been approved as a safe food preservative in China and the United States, and is widely used to preserve various foods, such as cooked meat products, fruit and vegetable juices, and egg products. Apart from its antibacterial properties, it also shows resistance to high temperatures, good solubility in water, and low toxicity [75]. The combination of ɛ-PL and carvacrol was synergistic against *E. coli* CICC 10664 and *S. aureus* CICC 21600, with fractional inhibitory concentration indices (FICI) of 0.375 and 0.5, respectively. Damage to cell membranes and a change in its permeability, as well as a decrease in the activity of the respiratory chain dehydrogenase, were observed [73].

The latest publication of Addo et al. describes the synergistic effect of a combination of carvacrol and cineole against *E. coli* ATCC 35150 growing on cucumbers. Strains of *E. coli* O157:H7 usually cause diarrhea, and they have a high capacity to form biofilms. The combination of carvacrol and cineole effectively inhibited the growth of *E. coli*, destroying the structure of the cell membrane and causing leakage of the bacterial cell contents. The ability to form a biofilm was also limited. In addition, the carvacrol–cineole mixture reduced the transcription of *E. coli* virulence genes, shiga toxin (*stx1*), intimin (*eae*), flagellar regulation (*flhD*), and quorum sensing gene AI-2 (*luxS*) [76].

In turn, Fan et al. [77] showed the synergistic effect of carvacrol and thermosonication in combating microorganisms infecting carrot juices. In the developed method, despite heating to 52 °C, the juices retained their original color and β-carotene content. In addition, the use of high-frequency thermosonication together with carvacrol allowed the shelf life of carrot juice to be extended by 25 days at 6 °C, while maintaining high sedimentation stability.

**Table 1:** Antimicrobial activity of carvacrol against strains of microorganisms.

Microorganism	Determination Method	MIC and/or MFC Value	References
*Aspergillus flavus* ATCC 204304	Serial dilution method in liquid medium	MIC: 0.098 mg/mL MFC: 0.098 mg/mL	[48]
*Candida albicans*	Serial dilution method in liquid medium	MFC: 256 mg/L	[37]
*Candida albicans* SC5314	Microdilution	MIC: 250 mg/L	[38]
*Candida albicans* SC5314	Microdilution	MIC: 247 μg/mL	[36]
*Candida auris*	Microdilution	MIC: 125 μg/mL	[38]
*Candida dubliniensis*	Serial dilution method in liquid medium	MFC: 161.3 mg/L	[37]
*Candida glabrata*	Serial dilution method in liquid medium	MFC: 238.9 mg/L	[37]
*Candida krusei*	Serial dilution method in liquid medium	MFC: 256 mg/L	[37]
*Penicillium* sp. isolated from soybeans	Serial dilution method in liquid medium	MIC: 0.098 mg/mL MFC: 0.98 mg/mL	[48]
*Saccharomyces cerevisiae*	Serial dilution method in liquid medium	MIC: <500 mg/L	[40]
*Bacterial strains*			
*Bacillus cereus* ATCC 14579	Double dilution method in liquid medium	MIC: 0.2 mg/mL	[61]
*Dickeya zeae* MS1	Serial dilution method in liquid medium	MIC: 0.1 mg/mL	[67]
*Enterococcus faecalis* isolated from French cheese	Serial dilution method in liquid medium	MIC: 0.625 mg/mL	[62]
*Enterococcus hirae* ATCC 10541	Serial dilution method in liquid medium	MIC: 312.5 μg/mL	[68]
*Escherichia coli* ATCC 25922	Microdilution	MIC: 0.225 mg/mL	[60]
*Escherichia coli* KBN10P03335	Serial dilution method in liquid medium	MIC: 150 μg/mL MBC: 300 μg/mL	[66]
*Gardnerella* sp. UM241	Microdilution	MIC: 0.08 μL/mL	[69]
*Klebsiella pneumoniae* CTX-M-8, OXA-48, KPC	Serial dilution method in liquid medium	MIC: 130 mg/L	[65]
*Pseudomonas aeruginosa* CIP 103467	Serial dilution method in liquid medium	MIC: 1.25 mg/mL	[62]
*Pseudomonas aeruginosa* ATCC 15442	Serial dilution method in liquid medium	MIC: 625 μg/mL	[68]
*Pseudomonas fluorescens* ATCC 13525	Double dilution method in liquid medium	MIC: 0.5 mg/mL	[64]
*Shewanella putrefaciens* ATCC 49138	Double dilution method in liquid medium	MIC: 0.5 mg/mL	[64]
*Staphylococcus epidermidis* ATCC 12228	Serial dilution method in liquid medium	MIC: <500 mg/L	[40]
*Staphylococcus aureus* ATCC 25923	Microdilution	MIC: 0.45 mg/mL	[60]
*Staphylococcus aureus* ATCC 6538	Double dilution method in liquid medium	MIC: 0.125 mg/mL	[64]
*Staphylococcus aureus* ATCC 6538	Serial dilution method in liquid medium	MIC: 78 μg/mL	[68]
*Streptococcus pyogenes* ATCC 19615, ATCC 49399	Serial dilution method in liquid medium	MBIC: 125 μg/mL MBEC: 250 μg/mL	[59]
*Vibrio parahaemolyticus* ATCC 17802	Double dilution method in liquid medium	MIC: 0.5 mg/mL	[64]

## 3. Antioxidant and Anti-Inflammatory Activity of Carvacrol

During normal cellular metabolism, free radicals or reactive oxygen intermediates are produced by cells. However, excessive accumulation of free radicals such as superoxide radicals, hydrogen peroxide, and hydroxyl radicals lead to tissue damage [3]. Infectious diseases often develop inflammation because the controlled accumulation of inflammatory factors such as leukotrienes, prostaglandins, TNF-α, or interleukins (e.g., IL-1, IL-6, IL-8, IL-10) has a beneficial effect in the fight against microbes. For example, cytokines are the link between cell damage and inflammation symptoms (cell migration, swelling, fever, or hyperalgesia). They are produced and released by many cell types in response to inflammatory stimuli [78]. It is worth emphasizing that the generally beneficial pro-inflammatory response, as a result of overstimulation, can contribute to the development of dangerous disease syndromes such as sepsis and septic shock [79,80]. For this reason, it is essential that the antimicrobial drug utilized also has antioxidant and anti-inflammatory activity. The antioxidant and anti-inflammatory activity of carvacrol is described in Table 2.

Carvacrol showed significant antioxidant activity in in vitro FRAP, DPPH, and TEAC assays, and Al-Mansori et al. [81] observed an increase in its activity with increasing compound concentration in the range of 50 to 1000 ppm. It is worth noting that carvacrol showed a stronger antioxidant effect than its isomer, thymol, and there was no synergistic effect found when using their mixture at the tested concentrations.

In studies conducted in guinea pigs, carvacrol concentrations of 120 and 240 μg/mL have been shown to reduce malondialdehyde levels compared to the control group [82]. Malondialdehyde is the main marker of polyunsaturated fatty acid peroxidation, as the breakdown of cell membrane integrity deactivates membrane proteins, receptors, and their associated enzymes, intensifying oxidative damage to cells [83]. It is worth emphasizing that in the concentrations utilized, the effect of carvacrol was comparable to that of dexamethasone—a glucocorticosteroid with strong and long-lasting anti-inflammatory, anti-allergic, and immunosuppressive effects [82]. A decrease in the level of malondialdehyde was also observed in the study by Carvalho et al. [84], where, in order to increase the bioavailability of carvacrol, it was encapsulated in solid lipid nanoparticles (SLN), and the effect of SLN on lung damage caused by inhalation of smoke through the respiratory tract was investigated. The study was conducted on 30 rats, which were sacrificed 24 h after inhalation trauma. SLN significantly reduced malondialdehyde levels compared to the control group [84]. Carvacrol significantly improves the level of available glutathione (GSH), which is mainly due to the removal of ROS. Carvacrol prevents lipid peroxidation by inducing SOD, GPx, GR, and CAT [85,86].

In an in vivo animal study, the administration of carvacrol at doses of 50–100 mg/kg was shown to have an anti-inflammatory effect, to relieve inflammatory edema in the paws of mice, and to reduce IL-1β and PGE2 levels. At the same time, they showed that the administration of a dose of 100 mg/kg reduces the expression of COX-2 and IL-1β mRNA. In turn, the level of IL-10 was increased by carvacrol [5,85,87]. Another study evaluated the anti-inflammatory effects of carvacrol in experimental models of edema induced by various phlogistic agents. In models of dextran or histamine-induced paw edema, carvacrol, at 50 mg/kg, reduced swelling by 46% and 35%, respectively. On the other hand, in the case of substance P-induced edema, carvacrol (100 mg/kg) reduced the edema by 46%. In addition, carvacrol reduced 12-*O*-tetradecanoyl phorbol acetate-induced ear swelling by 43% [88].

The anti-inflammatory properties of carvacrol were also tested in C57BL/6 mice administered 5% acetic acid rectally, thereby inducing epithelial changes in the intestinal mucosa and mimicking the phenotype of ulcerative colitis (UC), as ulcerative lesions of the distal colon or abnormalities of the intestinal crypts were formed that extended to the lamina propria. Prior to the induction of UC, rats were administered carvacrol at doses of 25, 50, or 100 mg/kg every 12 h for 3 days. Carvacrol significantly increased CAT, SOD, and GPx activity. In addition, reductions in abdominal hyperalgesia, colonic myeloperoxidase (MPO) activity, lipid peroxidation, and TNF-1 and IL-1 levels were observed [89,90].

The anti-inflammatory activity of carvacrol was tested in an in vitro model of streptococcal pharyngitis using human tonsil epithelial cells (HTonEpiCs) induced with *S. pyogenes* cell wall antigens. Pro-inflammatory cytokines such as IL-6, IL-8, ENA-78, and GCP-2 were found to decrease in a dose-dependent manner with carvacrol. In addition, HBD-2 production was significantly suppressed during the 24 h carvacrol treatment. Carvacrol also had an effect on PGE2 and COX-2 levels in cell suspensions [91].

Prostaglandins also play an important role in the inflammatory process. Cyclooxygenase (COX) is involved in the synthesis of these chemicals and has three isoforms, namely, COX-1, COX-2, and COX-3. COX-2 is rapidly expressed by extracellular stimuli as part of the inflammatory response, and is actually associated with inflammatory symptoms such as pain and fever [92]. The effect of carvacrol on the expression of the COX-2 gene in the LPS-stimulated macrophage cell line J774A.1 was studied. A decrease in COX-2 gene expression was found at carvacrol concentrations of 0.008% and 0.016% [93].

The effect of carvacrol on COX-2 has been studied in in vitro tests. Carvacrol (IC_50_ = 0.8 μM) inhibited the production of COX-2-catalysed prostaglandin E2, and was comparable to standard inhibitors such as indomethacin (IC_50_ = 0.7 μM) and NS-398 (N-[2-(cyclohexyloxy)-4-nitrophenyl]-methanesulfonamide; IC_50_ = 0.8 μM) [94].

The control of COX-2 expression is mediated by peroxisome proliferator-activated receptors (PPARs), which are ligand-dependent transcription factors belonging to the superfamily of nuclear receptors. PPAR-dependent suppression of COX-2 promoter activity by carvacrol was observed. This monoterpene is suggested to regulate COX-2 expression through its agonistic effect on PPARγ [95].

When male rats were given carvacrol at a dose of 20 mg/kg, it was found to positively modulate the Nrf2 transcription factor gene, which is an integral part of the host cell defense mechanism against oxidative stress. Nrf2 binds to antioxidant response elements (AREs) at the promoter sites of genes that encode several essential enzymes of the body’s anti-inflammatory response [96].

The compound that facilitates the colonization of host cells by bacteria through adhesion is lipopolysaccharide (LPS). It is the major component of the outer membrane of Gram-negative bacteria and is a foreign substance to higher organisms. However, LPS induces a rapid response in the immune system of the infected person. LPS is highly toxic when present in human blood, which is why it is often called an endotoxin. It causes fever and physical discomfort [97], and interacts with host cells, e.g., with monocytes, macrophages, polymorphonuclear leukocytes, and B and T lymphocytes, as well as various endothelial and epithelial cells. One of the pathways that triggers inflammation and is activated by LPS is the pathway with adapter proteins TRAM and TRIF, which are involved in the transduction of the LPS-derived signal. This pathway leads to the NF-κB-activated transcription of genes responsible for the production of proteins related to the body’s immune response [98]. LPS may also stimulate the inflammasome of NALP3, a high-molecular-weight multiprotein caspase-1-activating platform that coordinates the maturation of highly potent pro-inflammatory cytokines such as IL-1β and IL-18 [99]. The NALP3 inflammasome plays a key role in tissue damage caused by inflammation. In turn, TLR4 stimulation initiates the signaling pathway of bone marrow differentiation factor 88 (MyD88), which promotes rapid activation of NF-κB, consequently leading to an increase in IL-18, IL-6, IL-1β, tumor necrosis factor-α (TNF-α), and monocyte chemotactic protein-1 (MCP-1) [100]. It is worth noting that an overreaction of the infected body to LPS can result in immune suppression and organ dysfunction, which can even lead to death [101].

Lipopolysaccharide from *Porphyromonas gingivalis* (LPS-G), alone and in combination with carvacrol, was used in studies on the potential protective role of carvacrol in the inflammatory process. In an in vitro experiment, HL-1 cardiomyocytes were exposed to LPS-G and incubated with carvacrol. Carvacrol has been shown to have an anti-inflammatory effect by reducing the expression of TLR4, NF-κB, NALP3, and IL-1β. Therefore, the researchers suggest that carvacrol may provide a significant protective effect against the inflammatory stimulus generated by LPS-G, including suppression of the TLR4/NF-κB/NALP3/IL-1β signaling pathway [102].

In addition, studies conducted on mouse spleen cells have shown that carvacrol enhances anti-inflammatory cytokines, increasing the expression of interferon gamma (IFN-γ) and forkhead box P3 (FOXP3) genes while reducing pro-inflammatory activity and modifying the expression of IL-4 and IL-17 genes, as well as transforming growth factor beta (TGF-β) [103].

Carvacrol inhibited the activation of ERK1/2 by LPS. Carvacrol does not act at the extracellular level; at least, it does not affect the binding of LPS to TLR4/CD14. It is possible that carvacrol has a specific effect on the Ras/Raf pathway or that it acts on various membrane receptors that are also modulated by LPS. The pro-inflammatory effects of LPS are also mediated by NF-κB-dependent activation of transcription, and carvacrol blocks the effect of LPS on the translocation of NF-κB (p65) from the cytoplasm to the nucleus and its luciferase-coupled transcription. It has been shown to inhibit NF-κB transactivation even in the absence of LPS, demonstrating its role as an inhibitor of basal NF-κB activation in RAW 264.7 macrophages [104]. Activation of NF-κB by TLR4-mediated LPS is postulated to be dependent on TAK1, an upstream activator of both JNK and IKK [105], but studies by Somensi et al. have shown that carvacrol simultaneously inhibits ERK1/2 and NF-κB, suggesting an alternative pathway for its anti-inflammatory effect [104].

Another important signaling molecule besides LPS that plays an important role in the development of inflammatory immune responses is nitric oxide (NO) [25]. Carvacrol removed NO which had formed as a result of spontaneous degradation of sodium nitroprusside [106].

Carvacrol also affects heat shock proteins (Hsp), the overexpression of which is observed in inflammation caused by infection, high temperature, hypoxia, malnutrition, and exposure to chemicals or UV radiation, among other factors. HSPs are important factors regulating cell survival, differentiation, and death [107]. They prevent cellular damage and increase immunoregulation by activating anti-inflammatory T cells [108]. Carvacrol was able to co-induce the cellular expression of Hsp70 in Hsp70-specific T cell hybridomas in vitro. On the other hand, in in vivo studies on Peyer’s patches in mice, amplification of the T-cell responses to Hsp70 was observed after intragastric administration of carvacrol. Carvacrol administration also increased the number of CD4+CD25+FoxP3+ T cells, both systemically in the spleen and locally in the joint, and almost completely suppressed experimental proteoglycan-induced arthritis [109]. It is worth noting that Hsp70 interacts with the viral components of human cytomegalovirus, rabies virus, respiratory syncytial virus, human papilloma virus, and herpes simplex virus [107].

**Table 2:** Anti-inflammatory and antioxidant effects of carvacrol. (↑ increased or upregulate, ↓ decreased or downregulate).

Animal/Model	Doses of Carvacrol	Main Results	Reference
In vitro tests (ABTS, DPPH, FRAP, TEAC)	From 50 to 1000 ppm	Antioxidant activity	[81]
Guinea pigs exposed to cigarette smoke	120 and 240 μg/mL	Malondialdehyde↓	[82]
Inhalation of smoke in rats	Nanoparticles of carvacrol in form of SLN	Malondialdehyde↓	[84]
Induction of diabetes in rats	75 mg/kg for 8 weeks	SOD↓, GPx↓, Bax↓, Bcl-2↑, malondialdehyde↓	[86]
Mice	50–100 mg/kg	COX-2↓, IL-1β↓, PGE2↓, IL-10↑	[11]
C57BL/6 mice	25, 50, or 100 mg/kg	IL-1β↓, TNF-α↓, CAT↑, SOD↑, GPx↑	[89]
Model of streptococcal pharyngitis (HTonEpiCs)	4–125 µg/mL	IL-6↓, IL-8↓, ENA-78↓, GCP-2↓, HBD-2↓, PGE2↓, COX-2↓	[91]
LPS-stimulated cell line J774A.1	0.008% and 0.016%	COX-2↓	[93]
Ovine COX-2 activity assay	IC_50_ = 0.8 μM	Prostaglandin E2↓	[94]
Male Sprague–Dawley rats	20 mg/kg	Nrf2↑	[96]
HL-1 cardiomyocytes exposed to LPS-G	6.25–50 µM	IL-1β↓, TLR4↓, NFκ-B↓, NALP3↓	[102]
Mouse splenocytes	75–300 µg/mL	Gene expression of IL-4↓, IL-17↓, IFN-γ↓, FOXP3↓	[103]
RAW264.7 cells	0.2 mM	Hsp70↑	[109]

## 4. The Metabolism of Carvacrol

Carvacrol is generally considered to be a safe compound. Therefore, the lack of detailed data on its metabolism in humans is surprising. In a Phase I clinical trial, carvacrol was administered to healthy subjects at 1 and 2 mg/kg/day for 1 month, and no critical adverse reactions or clinically significant changes in biochemical, hematological, endocrine, renal, or hepatic function tests were observed [110]. More than 80% of carvacrol is absorbed or metabolized in the duodenum and stomach. Encapsulation of carvacrol in microcapsules of calcium alginate can effectively reduce its early absorption in the upper gastrointestinal tract after oral administration [111]. The involvement of CYP in the metabolism of carvacrol in humans has been confirmed in studies by Dong et al. [112] in 2012, when the compound was incubated with human liver microsomes (HLM) in the presence of NADPH. The CYP2A6 isoform was shown to be the major enzyme involved in the metabolism of carvacrol in humans. CYP1A2 and CYP2B6 isoforms also contributed to a lesser extent. In addition, 1-aminobenzotriazole (ABT), a non-specific CYP inhibitor, was found to almost completely inhibit the formation of carvacrol metabolites. Conversely, among the selective inhibitors of the CYP isoforms, 8-methoxypsoralen, a specific CYP2A6 inhibitor, significantly inhibited the formation of carvacrol metabolites, confirming that this isoform is primarily responsible for the metabolism of carvacrol in humans. The obtained results indicate that when carvacrol is co-administered with other drugs that are mainly metabolized by CYP2A6, further pharmacokinetic and clinical studies of these combinations of compounds should be performed [112]. Carvacrol may affect the toxicity of other compounds. CYP2E1 is involved in the metabolism of galactosamine. Significant increases in hepatic CYP2E1 mRNA and protein expression were found in rats intoxicated with d-galactosamine. In contrast, carvacrol supplementation suppressed mRNA expression of this protein, suggesting that carvacrol may have a remarkable hepatoprotective effect in the case of d-galactosamine poisoning [113].

**Figure 2:**
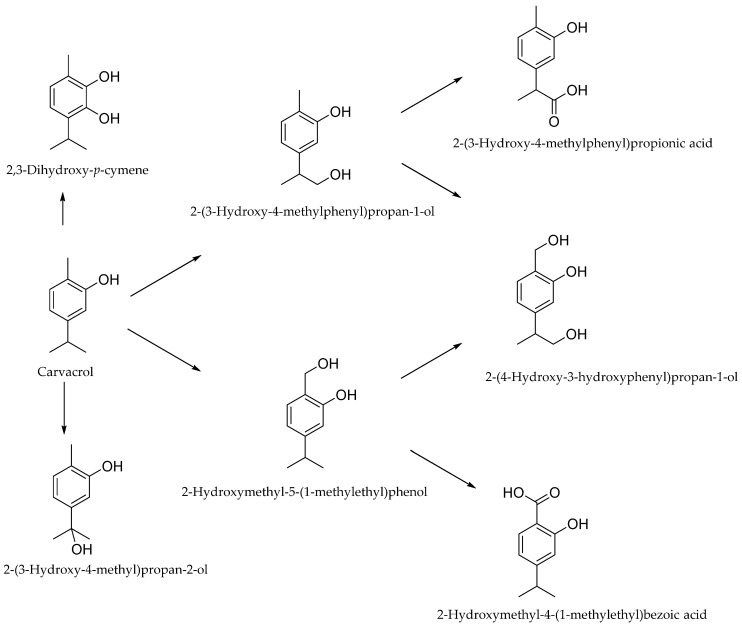
Metabolism and urinary excretion in rats.

There are more data on the metabolism of carvacrol in animals [109].In rats given oral carvacrol by gavage, the LD_50_ was 810 mg/kg body weight [114], while in mice, the LD_50_ was 80 mg/kg body weight after intravenous administration, 73.30 mg/kg body weight when administered intraperitoneally (i.p.), and 680 mg/kg body weight after subcutaneous administration. At very high doses (110–233.3 mg/kg bw), ataxia, decreased spontaneous motor activity, and somnolence were observed in mice before death [29,115]. In dogs, however, the lethal dose of intravenous carvacrol was 0.31 g/kg [116]. As early as 1932, it was found that carvacrol is rapidly excreted in the urine of rats and rabbits [117,118]. Carvacrol appears to be slowly adsorbed in the rabbit intestine, as 22 h after administration of 1.5 g, only about 25% of the dose was excreted in the urine [15]. More detailed studies on the metabolism of carvacrol were carried out by Austgulen et al. on Wistar rats in 1987. The compound (1 mmol/kg), dissolved in propylene glycol, was administered to the rats through a gastric tube. It was found to be excreted either unchanged or as glucuronides and sulfates. In much smaller amounts, hydroxylation of the methyl and isopropyl groups and further oxidation to the acid take place. Only traces of hydroxylation in the aromatic ring were observed, leading to the formation of 2,3-dihydroxy-*p*-cymene (Figure 2) [118,119].

## 5. Biotransformation of Carvacrol

Care for the environment forces the consideration of the impact of each antimicrobial agent on the surface water and soil ecosystem. For this reason, it is extremely important to determine the possible degradation pathways of each new antimicrobial drug, including carvacrol. Biotransformation processes are often the first stage of degradation, and are discussed in detail in this chapter.

**Figure 3:**
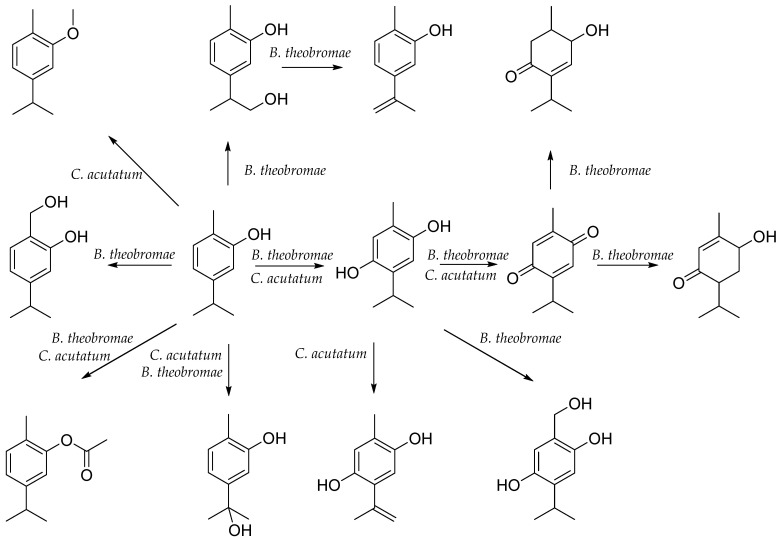
Biotransformation of carvacrol in plant culture.

The first reports on the biotransformation of carvacrol date back to 1972, when Herber et al. [120] transformed carvacrol into a hydroquinone derivative using a *Mucor hiemalis* culture. In addition, in their research, Numpaqe et al. [121] used the fungi *Colletotrichum acutatum* and *Botryodiplodia theobromae*, isolated from infected fruits of *Solanum betacea* (tamarillo) and *Persea americana* (avocado). In the culture of *C. acutatum*, the following were obtained: 2-methyl-5-isopropylhydroquinone (thymohydroquinone), 5-(1-hydroxy-1-methylethyl)-2-methylphenol, 2-isopropenyl-5-methylbenzene-1,4-diol, thymoquinone, and carvacrol ether derivative. In the culture of *B. theobromae*, 5-(2-hydroxy-1-methylethyl)-2-methylphenol, 2-hydroxymethyl-5-isopropylphenol, and 5-isopropenyl-2-methylphenol were observed. The transformation efficiency of *B. theobromae* for 5-(2-hydroxy-1-methylethyl)-2-methylphenol and 5-(1-hydroxy-1-methylethyl)-2-methylphenol was about 20%, and for the other compounds, it was much lower. It resulted that *C. acutatum* metabolized carvacrol much more quickly than *B. theobromae*, while the efficiency was only about 12% for the carvacrol ether derivative, and the values were lower for the other compounds [121] (Figure 3). In *Cladosporium* sp. culture, carvacrol was converted to 9-hydroxycarvacrol, which was then oxidized to carvacrol-9-oic acid [122].

## 6. Future Fields of Carvacrol Research

Due to its flavoring and antimicrobial properties, carvacrol is most often used in the food industry as a natural food preservative. An area of research that has recently been intensively developed is the possibility of using carvacrol as a component of active packaging, especially for food storage. Carvacrol placed on specific carriers or included in modified release preparations can limit the growth of pathogenic bacteria in food and slow down the process of its spoilage. Currently, research is also being carried out on the use of carvacrol as a feed additive in order to maintain the sensory and nutritional quality of poultry meat by inhibiting tissue lipid oxidation. It is debatable whether it is considered a safe antimicrobial compound despite the lack of modern research in this area. In particular, there are no unequivocal results indicating whether or to what extent carvacrol acts as an inducer or inhibitor of the CYP isoforms found in humans. Therefore, further research is needed in this area using modern methods, such as transcriptomic and proteomic techniques, metabolomics, X-ray crystallography, spectroscopy, and computer modeling. There are also no studies to date on the interaction of carvacrol with other food ingredients and drugs.

Although most scientific studies have shown significant potential for carvacrol as an antimicrobial agent, it is important to conduct appropriate clinical trials to determine the safety of the optimal doses and to further study their effectiveness against the strains of microorganisms that have been isolated from patients. Research is needed on the interaction of carvacrol with antibiotics, and in the search for such combinations of carvacrol with other drugs, researchers seek to prove that achieving a synergistic effect of both substances is possible.

In recent years, an intensively developed area of research in microbiology has been the structure and importance of microbial biofilms. In medicine, biofilm formation involving pathogenic strains is often a major therapeutic challenge, as such infections are extremely difficult to treat due to their natural tolerance to commonly used antibiotics and host immune responses. On the other hand, it is known that biofilms of non-pathogenic bacteria can effectively protect the body against infection with pathogenic strains. It is already known that carvacrol disrupts formed biofilms and prevents their formation. However, further research is needed on its mechanism of action on biofilms.

A serious challenge that stands in the way of the widespread use of carvacrol as an antimicrobial drug is its limited bioavailability, which results from unfavorable physicochemical properties such as high volatility, instability, and low solubility in water. Some studies have attempted to address this problem through the use of specific carriers or modified-release carvacrol formulations, which may result in improvements to the uptake of the active compound and its residence time in various organs or matrices. However, the systems developed so far, especially with the use of nanotechnology, require detailed toxicological studies in order to objectively assess the safety of their use.

Since carvacrol has a strong antimicrobial effect, it is very difficult to find strains of microorganisms capable of biotransforming this compound. Finding strains of microorganisms capable of transforming compounds with antimicrobial activity is particularly tedious and laborious, which may explain the very limited number of publications on this subject. However, research of this type is currently of particular importance in the context of a holistic view of the functioning of all chemical compounds in the environment.

## 7. Conclusions

Carvacrol is a naturally occurring compound that has achieved great importance, mainly due to its well-documented antimicrobial activity against many strains of bacteria, yeasts, and fungi (Table 1). The combination of the hydroxyl group and the delocalized electrons of the aromatic ring is essential for its activity. Importantly, the position of the hydroxy group is of less importance, as carvacrol often has comparable activity to its isomer, thymol. Carvacrol easily penetrates the cell membrane and can bind ATP or monovalent cations (e.g., K^+^), changing the cell membrane potential and influencing homeostasis. Changes in the respiratory chain and, consequently, decreases in ATP synthesis are also observed. Retained lipophilicity contributes to damage to the cell membrane of fungal and spore hyphae, changing the size and shape of cells, allowing for the leakage of cytoplasmic contents, and causing cell death. Carvacrol also disrupts formed biofilms and prevents their formation, especially in the case of *Candida*.

Serious infections are often accompanied by massive inflammation, which, in extreme cases, can lead to sepsis. Carvacrol prevents polyunsaturated fatty acid peroxidation by inducing SOD, GPx, GR, and CAT. It also contributes to the reduction in pro-inflammatory cytokines such as IL-6, IL-8, ENA-78, and GCP-2 in a dose-dependent manner. It also inhibits the synthesis of prostaglandins, affecting the cellular level of COX-2. It also reduces the level of the body’s immune response generated by LPS. Carvacrol also affects heat shock proteins (Hsp), which are overexpressed in infection-induced inflammation.

In the case of the antimicrobials utilized herein, it is important to understand the degradation pathways of the compounds in the environment. So far, it is known that strains of *B. theobromae*, *C. acutatum*, *M. hiemalis*, and *Cladosporium* sp. are capable of carvacrol biotransformation.

## Data Availability

Not applicable.
